# Commentary: Robust quantification of orientation selectivity and direction selectivity

**DOI:** 10.3389/fncir.2016.00025

**Published:** 2016-03-31

**Authors:** Tzvetomir Tzvetanov

**Affiliations:** Vision Research Laboratory, School of Life Sciences, University of Science and Technology of ChinaHefei, China

**Keywords:** neuronal data analysis, fitting, statistics, neuronal tuning, sampling

Mazurek et al. (2014) provided an important step forward in changing the old-fashioned manner of reporting orientation and/or direction selectivity of cells that involved the infamous OI–orientation index, DI, and related quantities such as orientation bias and direction bias (OB/DB, see Leventhal et al., 2003; OB/DB are the normalized or./dir. vector lengths, defined as *L*_*ori*_ and *L*_*dir*_ on p. 4 in Mazurek et al., 2014). At the beginning of their Results section, they demonstrated the unwanted features of these indexes, and showed that even non tuned cells can exhibit strong values of OI/DI. Therefore, they emphasized that little information was provided by these indexes (which I heartily welcome since it means I no longer have to discuss them with researchers and students) and investigated a statistical method of testing whether a cell is orientation- or direction-tuned.

In this commentary, first I would put forward what should be the first basic report in physiological studies: tuning characteristics. Second, I extend Mazurek et al.'s analysis and compare their proposed test to the fitting approach, which they only advocated for extracting tuning parameters. I conclude by mentioning the issue of tuning decision based on *p*-value and related questions.

First, OBs and associates cannot be the first analyses and reports since they are based on the tuning properties (see Figure 1 of Mazurek et al., 2014; and in my commentary, Figure 1A). Even when a cell is clearly tuned, these indexes have the highly unwanted feature of depending on at least two of the tuning characteristics: background rate, tuning width, and amplitude(s) (mentioned by the authors, pp. 9–10). Consequently, one compares data sets using unknowingly biased values, without clear interpretation of what changed in the tuning properties (see Figure 1B). As such, it is regrettable that the authors continue reporting results and statistical reliability with respect to OI/DI, when they should first have analyzed the tuning characteristics of the cells and how the decision criteria regarding the presence of tuning (e.g., their *T*^2^-test) depend on those parameters. Once the tuned cells are gathered and their parameters analyzed with respect to the hypotheses tested, then one may consider whether a composite index is appropriate for reporting the effects that are observed (e.g., SNR, OI, etc.).

**Figure 1:**
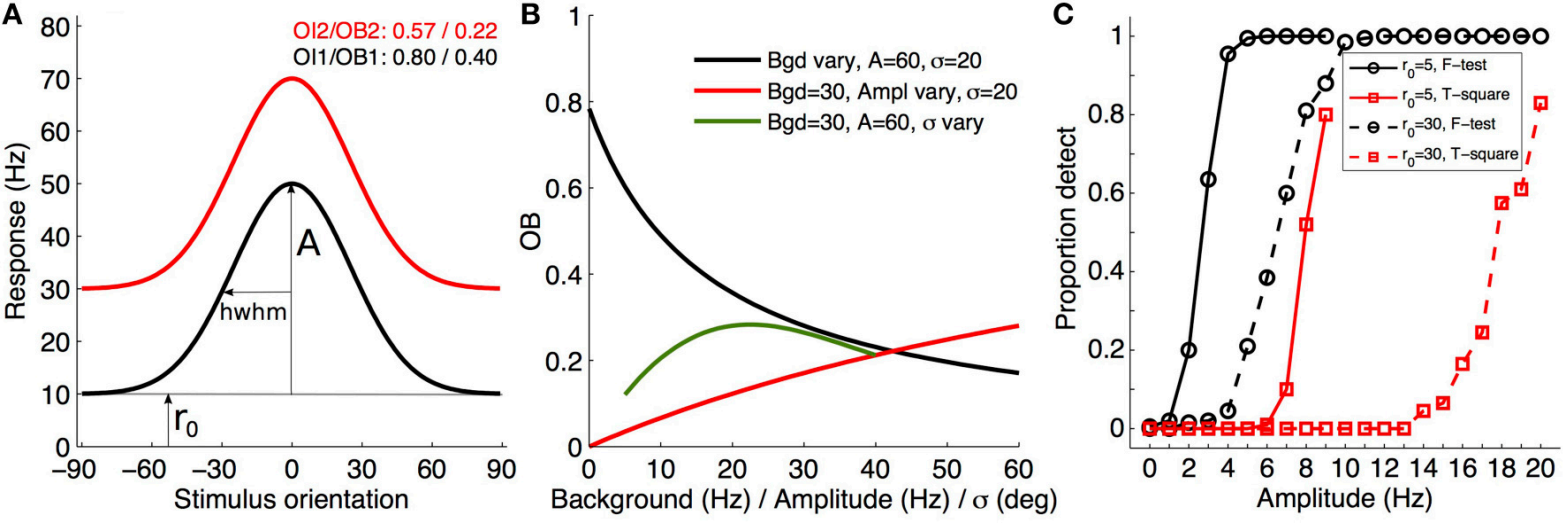
**Illustration of tuning, index variations, and statistical decision about tuning presence**. **(A)** Examples of two theoretical orientation-tuned cells only differing in background firing rate (r_0_ of 10 and 30 Hz; Gaussian curves with amplitudes of 40 Hz and σ of 25°) and their associated OI/OB (A, amplitude; hwhm, half-width at half-maximum). **(B)** Illustration of the variation of the orientation bias index (Leventhal et al., 1995) for Gaussian orientation-tuned cells when only one of the three parameters varies, with the two other fixed (see legend), demonstrating the difficulty of interpreting OI/OB variables without knowledge of the tuning parameters. **(C)** Proportion of detected tuned cells of a given amplitude (abscissa) when applying the Hotelling *T*^2^-test (red curves) or *F*-test (black curves), and for two different background firing rates (5 Hz in solid lines, 30 Hz in dashed lines). The model response was a von Mises direction-tuned cell (parameters: r_0_, a_1_ = 50 Hz, a_2_ = 0 Hz, *k* = 0.95, giving hwhm~32.6°; see Swindale, 1998), experimental sampling was every 15° (e.g., Schmolesky et al., 2000) with 10 repetitions per direction, and random noise around the mean was simulated as Poisson type. A total of 200 cells were simulated for each amplitude (each symbol in the plot) with random jitter of the preferred orientation across simulations within a 40° window. Each simulation was fitted with the von Mises two amplitude function (here unconstrained), and the statistical tests applied at α = 0.05/100 for multiple tests adjustment (Matlab code available on demand, or on http://www.researchgate.net/profile/Tzvetomir_Tzvetanov).

Second, the authors describe a method of testing whether a cell is tuned to orientation/motion direction (p.7). They propose creating sub-samples of single trial per orientation/direction from the measures, then computing the orientation/direction vector for each sub-sample and performing a Hotelling's *T*^2^-test to check whether the neuron's responses represent tuning against the hypothesis of uniform circular tuning. Later in their text (p. 11), they use fitting (Swindale, 1998), on those cells that were previously found to be tuned with the *T*^2^-test, to extract the tuning characteristics. Nevertheless, any model-based fitting approach allows one to test if a cell is tuned (H0, mean uniform response; and H1, the model describes the cell responses better than the mean) by using any relevant test statistics, for example an *F*-test between two nested models [*F* = (SS1 − SS2)/(df1 − df2)/(SS2/df2)]. Figure 1C plots the proportional results of detecting a direction tuning curve of a given amplitude using their proposed *T*^2^-test and the *F*-test, for two cells differing in background rate r_0_ (5 and 30 Hz; see legend and caption for details). While the sensitivities are strongly dependent on the tuning curve characteristics (but also on the experimental parameters, not shown), it is clear that their proposed Hotelling *T*^2^-test has a far worse sensitivity than the standard *F*-test (it is left to the reader to “try-and-see” other experimental or tuning parameters). Therefore, it would be advisable that any researchers first test various “presence of tuning” tests before settling for one in their analysis (out of the two considered here, one can think of the randomization test, the chi-square test that includes the errors in each measurement, or *R*^2^, for example). Then, they should clearly provide the reason for their choice in the Methods section of their report (e.g., Persi et al., 2011), e.g., the best sensitivity among the methods or the smallest false discovery rate.

As the authors nicely point out, it is important that the fitting procedure provides clearly interpretable parameters. I concur with the parameter constraint issues that they mention, and emphasize that researchers should consider in advance the sampling they will use in order to be able to make parameter estimates in the range of interest (e.g., if sampling steps were 22.5° it is difficult to believe that tuning widths at half-maximum of 45° or less would be easily measurable, that is, the curve depends on two or three data points for models with at least four parameters!).

Finally, the importance of their paper lies in the clear presentation of the necessity in the physiological field of using a proper criteria when deciding whether a cell is tuned to the investigated variable (orientation, motion, speed, spatial frequency, etc.). Thus, it is natural that a statistical test should be used and that users must take care about the issue of repeated tests with the associated α-level adjustment (Figure 1C, and caption). Such a decision criteria is firmly desirable in any report, and it remains to be seen how the recent decade of increasing importance in closely related scientific fields concerning *p*-value, decision methods, and replication issues (e.g., Open Science Collaboration, 2015; Halsey et al., 2016; Lazzeroni et al., 2016) will influence the field of neurophysiology.

## Author contributions

The author confirms being the sole contributor of this work and approved it for publication.

## Funding

The author is supported by the Fundamental Research Funds for the Central Universities (P.R.China).

### Conflict of interest statement

The author declares that the research was conducted in the absence of any commercial or financial relationships that could be construed as a potential conflict of interest.
